# An Overview of Interrater Agreement on Likert Scales for Researchers and Practitioners

**DOI:** 10.3389/fpsyg.2017.00777

**Published:** 2017-05-12

**Authors:** Thomas A. O'Neill

**Affiliations:** Individual and Team Performance Lab, Department of Psychology, University of CalgaryCalgary, AB, Canada

**Keywords:** interrater agreement, rwg, multilevel methods, data aggregation, within-group agreement, reliability

## Abstract

Applications of interrater agreement (IRA) statistics for Likert scales are plentiful in research and practice. IRA may be implicated in job analysis, performance appraisal, panel interviews, and any other approach to gathering systematic observations. Any rating system involving subject-matter experts can also benefit from IRA as a measure of consensus. Further, IRA is fundamental to aggregation in multilevel research, which is becoming increasingly common in order to address nesting. Although, several technical descriptions of a few specific IRA statistics exist, this paper aims to provide a tractable orientation to common IRA indices to support application. The introductory overview is written with the intent of facilitating contrasts among IRA statistics by critically reviewing equations, interpretations, strengths, and weaknesses. Statistics considered include *r*_wg_, $r_{\text{wg}}^{*}$, *r*′_wg_, *r*_wg(p)_, average deviation (*AD*), *a*_wg_, standard deviation (*S*_wg_), and the coefficient of variation (*CV*_wg_). Equations support quick calculation and contrasting of different agreement indices. The article also includes a “quick reference” table and three figures in order to help readers identify how IRA statistics differ and how interpretations of IRA will depend strongly on the statistic employed. A brief consideration of recommended practices involving statistical and practical cutoff standards is presented, and conclusions are offered in light of the current literature.

## Introduction

The assessment of interrater agreement (IRA) for Likert-type response scales has fundamental implications for a wide range of research and practice. One application of IRA is to quantify consensus in ratings of a target, which is often crucial in job analysis, performance assessment, employment interviews, assessment centers, and so forth (e.g., Brutus et al., 1998; Lindell and Brandt, 1999; Walker and Smither, 1999; Morgeson and Campion, 2000; Harvey and Hollander, 2004). Another application of IRA is to determine the appropriateness of averaging individual survey responses to the group level (van Mierlo et al., 2009). In that spirit, IRA has been used to support the aggregation of individual ratings to the team level, follower ratings of leadership to the leader level, organizational culture ratings to the organizational level, and leadership ratings to the leader level (see discussions by Rousseau, 1985; Chan, 1998; Kozlowski and Klein, 2000). If consensus in the ratings of a target is low, then the mean rating may be a misleading or inappropriate summary of the underlying ratings (George, 1990; George and James, 1993). Underscoring the importance of IRA statistics is that, unlike interrater reliability and consistency statistics, IRA provides a single value of agreement for each rating target, thereby facilitating identification of units of raters who are very high or very low in agreement. This advantageous feature also permits subsequent investigation of other substantive and theoretically interesting variables that may be related to variance in agreement (Klein et al., 2001; Meade and Eby, 2007), or as a moderator of predictor-criterion relations (e.g., climate strength; Schneider et al., 2002).

IRA are particularly common when collecting systematic observations of behavior or phenomena. For example, Bernardin and Walter (1977) found that training and diary keeping reduced the errors in performance ratings. O'Neill and Allen (2014) investigated subject-matter experts' ratings of product innovation. Weingart et al. (2004) observed and coded negotiation behavior between teams and reported on methods for doing so. Many more examples exist. The key is that IRA becomes highly relevant when judges observe and provide ratings of behavior or phenomena, and the absolute agreement of those ratings is of interest.

Despite the widespread application of IRA statistics and the extensive research focusing on IRA, it appears that considerable challenges persist. For example, a recent review by Biemann et al. (2012) identified situations in which applications of IRA for aggregation of leadership ratings has been misused, as ratings were aggregated (or not) based on flawed interpretations of IRA. A possible contributing factor of the potential for IRA misuse is that considerations of the logic underlying equations and interpretations of alternative IRA statistics have been relatively scattered across organizational (e.g., Lindell and Brandt, 1999), methodological (e.g., Cohen et al., 2001), and measurement (e.g., Lindell, 2001) journals, thereby making it difficult for researchers and practitioners to contrast the variety of statistics available and to readily apply them appropriately. LeBreton and Senter (2008) provided a seminal review of IRA and consistency statistics, but the focus was largely on implications of these types of statistics for multilevel research methods and not on the many other applications of IRA (e.g., agreement in importance ratings collected in job analysis; Harvey, 1991). Elsewhere, IRA statistics have been investigated as dispersion measures of substantive constructs in multilevel research in terms of criterion validity (Meade and Eby, 2007), power (e.g., Roberson et al., 2007), significance testing (e.g., Cohen et al., 2009; Pasisz and Hurtz, 2009), and performance under missing data conditions (Allen et al., 2007; Newman and Sin, 2009). Importantly, some existing articles may be seen as highly technical for some scholars that are new to the IRA literature (e.g., Lindell and Brandt, 1999; Cohen et al., 2001), and other reviews tend to focus on only one or two IRA statistics (e.g., Castro, 2002).

Given the above, what is needed is a relatively non-technical and tractable orientation to IRA that facilitates comparison and interpretation of various statistics for scholars. Accordingly, the purpose of this article is to contribute by providing an accessible and digestible IRA resource for researchers and practitioners with a diverse range of training and educational backgrounds who need to interpret or report on IRA. The current article fills a gap by reporting on an introductory comparative analysis involving eight IRA statistics: *r*_wg_, $r_{\text{wg}}^{*}$, *r*′_wg_, *r*_wg(p)_, average deviation (*AD*), *a*_wg_, standard deviation (*S*_wg_), and the coefficient of variation (*CV*_wg_). A unique contribution is a “quick reference” table containing citations, formulas, interpretations, strengths, and limitations (see Table 1). The aim of Table 1 is to support expedient consideration of the appropriateness of various IRA statistics given a researcher or practitioner's unique situation, and to serve as a foundation for more focused, complex issues addressed in technical guides (e.g., Burke and Dunlap, 2002). Further, three figures attempt to clarify the behavior of IRA statistics and to supplement understanding and interpretation of various IRA statistics. The article introduces James et al.'s (1984) *r*_wg_, some potential issues with interpretations of that statistic, and numerous contemporary alternatives. Before beginning, a comment on IRA and interrater consistency is offered.

**Table 1:** **Summary of interrater agreement statistics for likert-type response scales**.

**Statistic (citations)**	**Formula**	**Interpretation**	**Strengths**	**Limitations**
*r*_wg_ (James et al., 1984; see also Finn, 1970)	1 – (*S*_x_^2^/σ_eu_^2^) *S*_x_^2^ = observed variance in judges' ratings on the single item; and σ_eu_^2^ = variance of the rectangular, uniform null distribution, (*A*^2^ – 1)/12, where *A* is the number of discrete Likert-type response options.	A value of 1.0 indicates complete agreement. A value of 0 indicates agreement equal to the null distribution (i.e., one index of completely random responding. Values below 0 or above 1.0 are assumed to be the result of sampling error and should be reset to 0 (see James et al., 1984).	Commonly used in the literature and generally known to researchers and reviewers. Likely the most researched agreement statistic. Linear function facilitates interpretation.	Uniform distribution may inappropriately model random responding, and selecting an alternative null distribution can be difficult (for guidance, see LeBreton and Senter, 2008). May not be directly comparable (i.e., equivalent) across different means of group ratings, number of raters, or sample sizes. It is not uncommon for values to exceed +1.0 or fall below 0. These inadmissible values might not be the result of sampling error. Resetting the values to 0 may therefore be inappropriate and result in loss of information (Brown and Hauenstein, 2005).
*r*_wg(j)_ (James et al., 1984)	$\frac{J(1-\bar{{S_{\text{x}}}^{2}}/{\sigma_{\text{eu}}}^{2})}{J(1-\bar{{S_{\text{x}}}^{2}}/{\sigma_{\text{eu}}}^{2})+(\bar{{S_{\text{x}}}^{2}}/{\sigma_{\text{eu}}}^{2})}$ *J* = number of items; *S*_x_^2^ = mean of the observed variance in judges' ratings on each scale item; and $\sigma_{eu}^{2}=$ see above.	A value of 1.0 indicates complete agreement. A value of 0 indicates agreement equal to the null distribution. Values below 0 or above 1.0 are assumed to be the result of sampling error and should be reset to 0 (see James et al., 1984).	Commonly used in the literature and generally known to researchers and reviewers. Likely the most researched agreement statistic.	Same as *r*_wg_, above. May not be directly comparable (i.e., equivalent) across different means of group ratings or the number of raters. It is upwardly influenced by the number of discrete Likert scale response options. Values in between 1.0 and 0 are difficult to interpret because the function is non-linear.
r^*^_wg_ (Lindell and Brandt, 1997)	1 – (*S*_x_^2^/σ_eu_^2^) or 1 – (*S*_x_^2^/ σ_*mv*_^2^) *S*_x_^2^ = see above; σ_eu_^2^ = see above; and $\sigma_{mv}^{2}$ = variance of the maximum dissensus distribution, 0.5(X^2^_*U*_ + X^2^_*L*_) – [0.5(X_*U*_ + X_*L*_)]^2^	If using σ_eu_^2^, the interpretation is the same as *r*_wg_, described above. If using σ_*mv*_^2^, a value of 1.0 indicates complete agreement;.5 indicates agreement equal to the uniform null distribution; and 0 indicates theoretical maximum dissensus. r^*^_wg_ using σ_*mv*_^2^ will tend to be greater than is r^*^_wg_ using σ_eu_^2^ and *r*_wg_ will always be less than is r^*^_wg_. Values below 0 (using σ_eu_^2^) and below 0.5 (using σ_*mv*_^2^) are possible when agreement is low (i.e., it suggests bimodal distributions).	Presents a compelling alternative to the uniform null distribution (σ_eu_^2^) by positing the theoretical maximum dissensus (σ_*mv*_^2^) for use as a random error term. Circumvents problems of inadmissible values by allowing for meaningful interpretations when *S*_x_^2^ it exceeds σ_eu_^2^.	May not be directly comparable (i.e., equivalent) across different means of group ratings. Maximum dissensus may inappropriately model random responding, and selecting an alternative null distribution can be difficult (for guidance, see LeBreton and Senter, 2008). May be positively correlated with group mean extremity.
r^*^_wg_*_(*j*)_* (Lindell et al., 1999)	r^*^_wg_*_(*j*)_* = 1 – (S̄_x_^2^/ σ_eu_^2^) or r^*^_wg_*_(*j*)_* = 1 – (S_x_^2^/ σ_*mv*_^2^) *S*_x_^2^ = see above; $\sigma_{eu}^{2}=$ see above; and σ_*mv*_^2^ = see above.	Same as r^*^_wg_, above.	Same as r^*^_wg_, above. With increasing items the function remains linear, unlike *r*_wg(_*_*j*_*_)_.	Same as r^*^_wg_, above.
*r'*_wg(_*_*j*_*_)_ (Lindell, 2001)	1 – (*S*_*y*_^2^/σ_*eu*_^2^) *S_y_*^2^ = variance of individual judges' scale means; and σ_eu_^2^ = see above.	Less attenuated than is r^*^_wg_*_(*j*)_* with σ_eu_^2^ relative to *r*_wg(_*_*j*_*_)._ Interpretation is otherwise similar to r^*^_wg__(_*_*j*_*_)_.	Less attenuated than is r^*^_wg_; Otherwise the strengths are the same as those of r^*^_wg__(_*_*j*_*_)_.	Shares many of the same limitations as does r^*^_wg_*_(*j*)_*except *r'*_wg(_*_*j*_*_)_ will often be less attenuated. Application has been rare in the literature and, accordingly, researchers and reviewers may be unaware of the underlying logic.
*r_wg(p)_* (LeBreton et al., 2005; LeBreton and Senter, 2008)	Identify subgroups, calculate each subgroup's agreement score, check homogeneity of variances and, if supported, substitute sample-weighted average group variance (denoted *S*^2*x*.τ^) value into *r*_wg_or *r*_wg__(_*_*j*_*_)_equation. Homogeneity of variances can be tested using Fisher's *F*-test by dividing the larger subgroup variance by the smaller subgroup variance, which is approximately distributed as the F distribution with degrees of freedom for subgroup 1/degrees of freedom for subgroup 2 (see Crawley, 2007, p. 289 for application in *R*).	Has same interpretation as does previous *r*_wg_conceptualization except considers subgroup agreement differences by averaging them.	Allows for consideration of theoretically meaningful subgroups. Addresses limitation of inadmissible values that can be problematic for *r*_wg_ and *r*_wg(_*_*j*_*_)_.	Has many of the same interpretational problems as do previous *r*_wg_statistics reviewed (e.g., difficulties in choosing an appropriate null distribution). Can be difficult to generate theoretical predictions *a priori* about the existence of subgroups. Assumes homogeneity of subgroup variances. If homogeneity assumptions cannot be supported, separate *r*_wg_ values based on subgroups could be another option.
*AD_*M*_*_(_*_*j*_*_)_ (Burke et al., 1999; Burke and Dunlap, 2002)	∑(|*x*_*i*_– $\bar{x}$|)/*k* *x*_*i*_ = a judge's rating on the item; $\bar{x}$= is the group mean rating on the item; and *k* is the number of judges.	Indexes the average distance of judges' ratings from the group's scale mean. Considerable justification for practical cutoff criteria have been proposed, but they are not without assumptions (see Section Standards for Agreement).	Interpretation is not complicated by changes (e.g., non-linearity) in the number of Likert categories (bearing in mind greater deviations are expected given category increases). Circumvents problems associated with choosing an appropriate null distribution.	May be negatively correlated with group mean extremity. Does not permit explicit modeling of random responding (i.e., has no null distribution term). AD values are highly dependent on the number of scale categories employed. This makes it very difficult to compare AD values of scales differing in length.
*AD_*M*_*_(_*_*J*_*_)_ (Burke et al., 1999; Burke and Dunlap, 2002)	∑*AD_*M*_*_(_*_*j*_*_)_/*J* *J* = see above.	Shares interpretations of *AD_*M*_*_(_*_*j*_*_)_ except generalizes to multi-item scales.	Same advantages as *AD_*M*_*_(_*_*j*_*_)_. Takes the average of each *AD_*M*_*_(_*_*j*_*_)_ and, therefore, does not unnecessarily complicate the multi-item interpretation.	Same limitations of *AD_*M*_*_(_*_*j*_*_)_.
*a*_wg(__1__)_ (Brown and Hauenstein, 2005)	1 – [(2 ^*^S_*x*_^2^)/*S_mpv/m_*^2^] *S*_x_^2^ = see above, and *S*_mpv/m_^2^ [(H+L)*M*–(*M*^2^) – H^*^L)^*^[*k*/(*k*-1)] where H = maximum discrete scale value; L = minimum discrete scale value; *M* = observed mean rating; and *k* = number of raters.	A value of +1.0 indicates perfect agreement, given the group mean. A value of 0 indicates the observed variance is 50% of the maximum variance, given the group mean. A value of −1.0 indicates maximum disagreement given the group mean. Will equal single-item *r*_wg_ when the group mean is at the scale mid-point and the variance equations (sample vs. population) are not mismatched for *r*_wg_. Will equal single and multi-item r^*^_wg_ using σ_eu_^2^ when the group mean is at the midpoint and the variances are not mismatched.	Controls for the extremeness of the group mean by not relying on a single specification of the null distribution. Uses the unbiased, sample variance to calculate observed and theoretical random variance terms, whereas the *r*_wg_ family of statistics confound these. Circumvents problems of inadmissible values. Will not be affected by sample size because it employs matched variances.	Requires at least *A*-1 raters for calculating interpretable *a*_wg_ values, where *A* is equal to the number of Likert response categories (see Brown and Hauenstein, 2005). Is not interpretable at face value beyond certain extreme group means. That is, the minimum mean with interpretable *a*_wg_ = [*L*(*k*-1) + H]/*k*; and the maximum mean with interpretable *a*_wg_ = [*H*(*k*-1) + L]/*k*.
*a*_wg(_*_*j*_*_)_ (Brown and Hauenstein, 2005)	∑*a*_wg(__1__)_/*J* *J* = see above.	Shares interpretations of *a*_wg(__1__)_, except generalizes to multi-item scales.	Same advantages as *a*_wg(__1__)_. Takes the average of each *a*_wg(__1__)_ and, therefore, does not unnecessarily complicate the multi-item interpretation.	Same limitations as *a*_wg(__1__)_.
*S*_wg_ (Schmidt and Hunter, 1989)	{[∑ (*x*_*i*_ – $\bar{x}$)^2^]/(n – 1)}^1/2^ *x*_*i*_ = a judge's rating on the item, $\bar{x}=$ the group mean rating on the item; and *n* is the number of group members.	The root of the average squared judge deviation from the mean.	Provides a straightforward and direct index of agreement.	Will be scale dependent such that a greater number of response options will tend to produce greater *S*_wg_. Does not permit explicit modeling of random responding (i.e., has no null distribution term).
*S*_wg(_*_*j*_*_)_	∑*S*_wg_/*J* *J* = see above.	Shares interpretations of *a*_wg(__1__)_, except generalizes to multi-item scales.	Same advantages as *S*_wg_. Takes the average of each *S*_wg__(__1__)_ and, therefore, does not unnecessarily complicate the multi-item interpretation.	Same limitations as *S*_wg_.
*CV*_wg_ (Allison, 1978; Bedeian and Mossholder, 2000)	*S*_wg_/$\bar{x}$ s = see above; and $\bar{x}$= see above.	Rescales the standard deviation by taking into account the mean. Large values suggest large variance relative to the mean (and scale).	Samples with larger means may be expected to have greater standard deviations than samples with smaller means. The *CV*_wg_ will not be affected by the scale mean, thereby facilitating comparisons across samples (i.e., groups) with different means (and scaling).	It is difficult to decide what constitutes high and low consensus based on *CV*_wg_ values; therefore, application and interpretation of *CV*_wg_ may be difficult. The assumption of a non-negative ratio scale may not always be tenable. The *CV*_wg_ is intended for situations in which means vary widely. If groups tend not to differ much on sample means there is little reason to adopt *CV*_wg_. Does not permit explicit modeling of random responding (i.e., has no null distribution term).
*CV*_wg_(*_*j*_*_)_	∑*CV*_wg_/*J* *J* = see above.	Shares interpretations of *CV*_wg_, except generalizes to multi-item scales.	Same advantages as *CV*_wg_.	Same disadvantages as *CV*_wg_.

## James et al.'s IRA: *r*_wg_ for single and multiple items

### General logic

For use on single-item scales, James et al. (1984; see also Finn, 1970) introduced the commonly-used, and perhaps most ubiquitous, IRA statistic known as *r*_wg_. This statistic is a function of two values: the observed variance in judges' ratings (denoted as $S_{\text{x}}^{2}$), and the variance in judges' ratings if their ratings were random (denoted as $\sigma_{\text{e}}^{2}$ in its general form, referred to as the *null distribution*). What constitutes a reasonable standard for random ratings is highly debated. One option, apparently the default in most research, is the rectangular or uniform distribution calculated with the following (Mood et al., 1974):

(1) $$\sigma_{\text{eu}}^{2}=(A^{2}-1)/12$$

where *A* is the number of discrete Likert response alternatives. This distribution yields the variance obtained if each Likert category had an equal probability of being selected. Observed variance in judges' ratings on a single item can be compared to this index of completely random responding to determine the proportion of error variance present in the ratings:

(2) proportion of random variance in judges' ratings=Sx2/σeu2

If this value—the proportion of error variance in judges' ratings—is subtracted from 1, the remaining variance can be interpreted as the proportion of variance due to agreement. Hence, the IRA for single item scales can be:

(3) $$r_{\text{wg}}=1-(S_{\text{x}}^{2}/\sigma_{\text{eu}}^{2})$$

Whereas, Equation (3) is for single-item scales, James et al. (1984) derived an index for multi-item response scales denoted as *r*_wg(*j*)_. It applies the Spearman-Brown prophecy formula (see Nunnally, 1978) to estimate IRA given a certain number of scale items (although James et al., 1984 did not use the Spearman-Brown in its derivation; see also LeBreton et al., 2005). Further, the term $S_{x}^{2}$ from Equation (3) is substituted with the mean $S_{x}^{2}$ derived from judges' ratings on each scale item to yield the following:

(4) $${\text{r}}_{\text{wg}(\text{j})}=J(1-\bar{S_{x}^{2}}/\sigma_{\text{eu}}^{2})/[J(1-\bar{S_{x}^{2}}/\sigma_{\text{eu}}^{2})+(\bar{S_{x}^{2}}/\sigma_{\text{eu}}^{2})]$$

where $\sigma_{\text{eu}}^{2}$ is the same as in Equation (1), and *J* is the number of items.

### Interpretation

**Figure 1:**
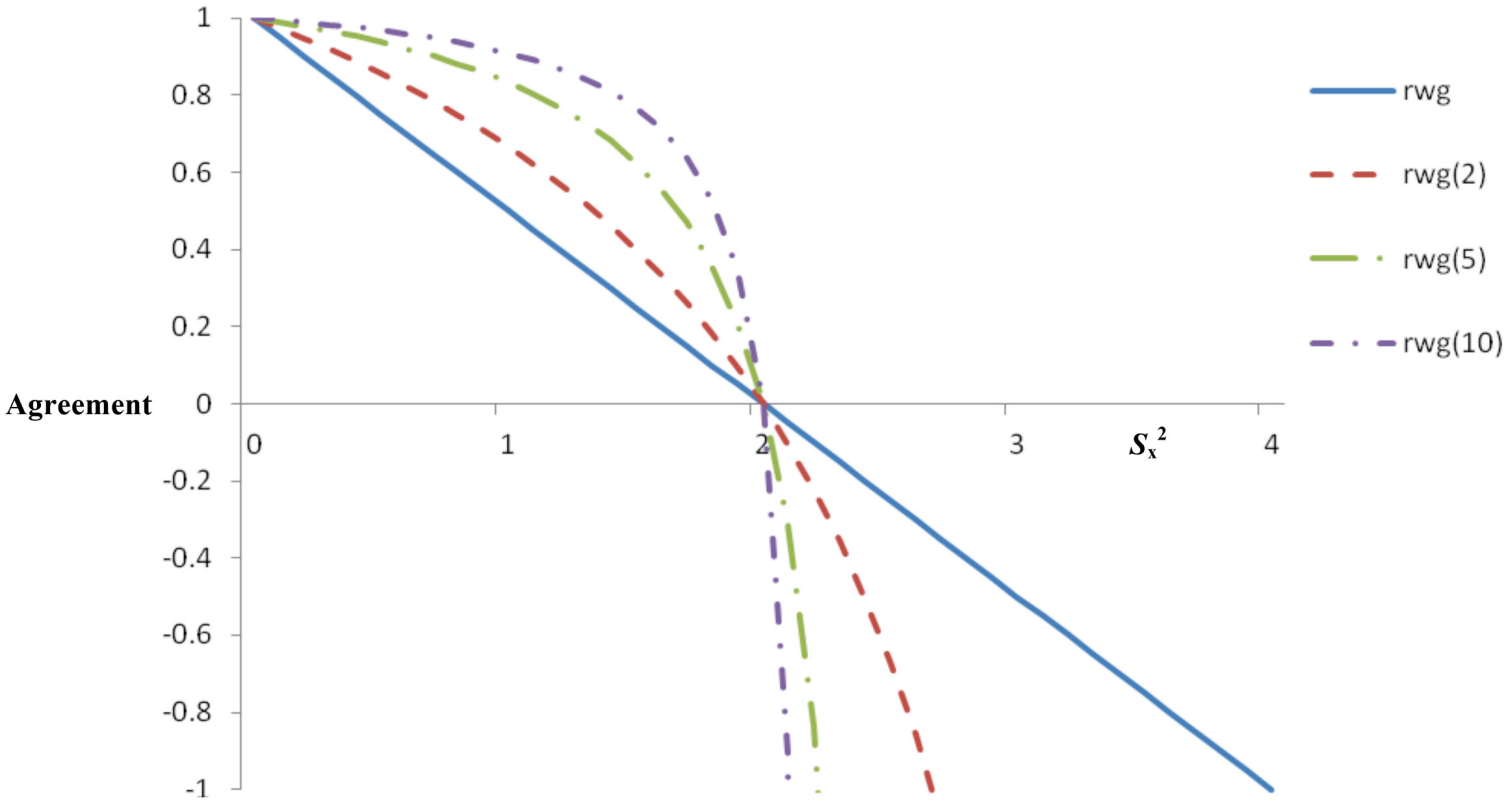
**Single and multiple-item ***r***_**wg**_ across levels of ***S***_x_^2^ (five-point scale)**.

Figure 1 shows the range of *r*_wg_ values across all possible levels of mean $S_{x}^{2}$ based on four raters and a five-point Likert scale (see also Lindell and Brandt, 1997). One observation from Figure 1 is that the single-item *r*_wg_ is a linear function, such that complete agreement equals 1.0 and uniform disagreement equals 0 (i.e., raters select response options completely at random). But notice that for *S*_x_^2^ > 2—that is, where *S*_x_^2^ exceeds $\sigma_{\text{eu}}^{2}$ − *r*_wg_ takes on negative values. Figure 1 also contains the *r*_wg(*j*)_ function ranging from −1.0 to +1.0 across levels of *S*_x_^2^ based on four raters, a five-point Likert scale, and two, five, and ten items. Consistent with expectations, when *r*_wg(*j*)_ is 1.0 agreement is perfect and when *r*_wg(*j*)_ is 0 there exists uniform disagreement. However, at all other levels of *r*_wg(*j*)_, interpretation is complicated because the shape of the function changes depending on the number of items. Consider that, as the mean *S*_x_^2^ moves from 0 to 1.5, *r*_wg(*j*)_ ranges from 1.0 to 0.40 [*r*_wg(2)_], 1.0 to 0.63 [*r*_wg(5)_], and 1.0 to 0.77 [*r*_wg(5)_] suggesting that *r*_wg(*j*)_ is insensitive to substantial changes at reasonable levels of mean $S_{\text{x}}^{2}$, and it might imply surprisingly high agreement even when there is considerable variance in judges' ratings. This also illustrates the extent to which the problem increases in severity as the number of items increases. The pattern creates the potential for misleading or inaccurate interpretations when the shape of the function is unknown to the researcher. Another issue is that *S*_x_^2^ > 2 produces inadmissible values that are outside the boundaries of *r*_wg(*j*)_ (i.e., < 0 or > 1.0). Regarding inadmissible values, James et al. (1984) suggested that these may be a result of sampling error. Other possible contributing factors include inappropriate choices of null distributions and the existence of subgroups. One recommended procedure is to set inadmissible values to 0 (James et al., 1993). This could be an undesirable heuristic, however, because it results in lost information (Lindell and Brandt, 1999, 2000; Brown and Hauenstein, 2005).

### Potential cause for concern

Whereas, *r*_wg_ is arguably the most widely used IRA statistic, there are five issues concerning its interpretation. First, there is the issue of non-linearity described above. This non-linearity, occurring with increased magnitude as the number of scale items increases, renders interpretations of agreement levels ambiguous compared to interpretations of linear functions. The appropriateness of interpretations may be particularly weak if the researcher or practitioner is unaware that the function is non-linear. Indeed, scales with a large number of items will almost always have very high agreement (Brown and Hauenstein, 2005; cf. Lindell and Brandt, 1997; Lindell et al., 1999; Lindell, 2001), which limits the interpretational and informative value of *r*_wg(*j*)_ with scales containing more than a few items. Figure 1 clarifies this. Second, there are difficulties involving inadmissible values, also described above. Resetting these values to 0 or 1.0 seems suboptimal because potentially useful information is arbitrarily discarded. It would be advantageous if that additional information could be used to further shed light on agreement. Third, *r*_wg_ and *r*_wg(*j*)_ appear to be related to the mean rating extremity. Brown and Hauenstein (2005) found a correlation between mean judge ratings and *r*_wg(*j*)_ values of 0.63. This is not surprising because mean ratings falling closer to the scale endpoint must have restricted variance (i.e., agreement). Thus, *r*_wg_ will be affected by the mean rating. Fourth, the typical selection of σ_e_^2^, the theoretical distribution of random variance, seems to be the rectangular distribution, described above as σ_eu_^2^ (Cohen et al., 2009). But the σ_eu_^2^ uses scaling that leads to inadmissible values (i.e., *r*_wg_ < 0), and other distributions may be an improvement (LeBreton and Senter, 2008). Whereas, James et al. (1984) offered alternatives to σ_eu_^2^ that attempt to model response tendencies or biases, in many cases it is difficult to make a choice other than $\sigma_{\text{eu}}^{2}$ that can be defended (for laudable attempts, see Kozlowski and Hults, 1987; LeBreton et al., 2003). One alternative to $\sigma_{\text{eu}}^{2}$, suggested by Lindell and Brandt (1997), however, seems promising (described further below). Fifth, the observed variance in the numerator, *S*_x_^2^, tends to decrease with sample size, which creates the potential to spuriously increase *r*_wg_ (Brown and Hauenstein, 2005).

Given the above issues involving James et al.'s (1984) *r*_wg_, the remainder of this article describes some alternatives and how each alternative was proposed to address at least one of the issues raised. Knowledge of this is intended to help the researcher or practitioner make informed decisions regarding the most applicable statistic (even *r*_wg_) given his or her unique situation.

## $r_{wg}^{*}$ with the rectangular null and maximum dissensus null distributions

### General logic

**Figure 2:**
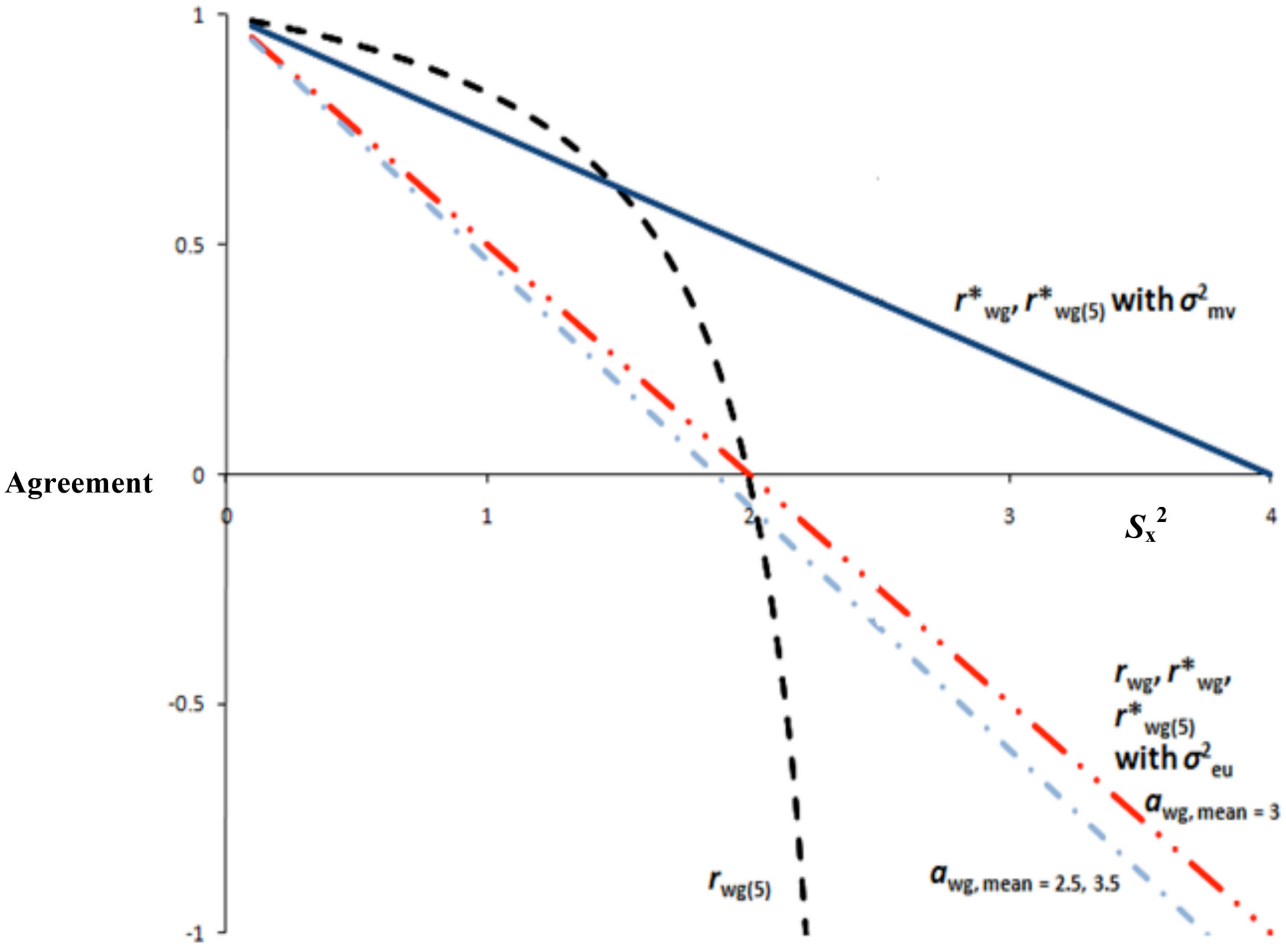
**Sample ***r***_**wg**_-family and ***a***_**wg**_ statistics across levels of ***S***_x_^2^ (five-point scale)**.

In order to overcome shortcomings of non-linearity and inadmissible values of *r*_wg_ and *r*_wg(*j*)_, Lindell et al. (1999) proposed $r_{\text{wg}}^{*}$. $r_{\text{wg}}^{*}$ using σ_eu_^2^ is equal to *r*_wg_ except $r_{\text{wg}}^{*}$ allows for meaningful negative values to −1.0. Negative values will occur when $S_{x}^{2}$ exceeds the variance of the rectangular distribution, $\sigma_{\text{eu}}^{2}$, and these negative values indicate bimodal distributions. In other words, clusters of raters are at or near the scale end points. Unlike *r*_wg_, which does not consider negative values to be admissible, $r_{\text{wg}}^{*}$ recognizes that this information can provide theoretical insight into the nature of the disagreement. $r_{\text{wg}(j)}^{*}$ with $\sigma_{\text{eu}}^{2}$ also uses the same equation as does *r*_wg_ but instead uses the mean variance in the numerator:

(5) $$r_{\text{wg}}^{*}=1-(\bar{{S_{\text{x}}}^{2}}/\sigma_{\text{eu}}^{2})$$

where $S_{x}^{2}$ is the mean of the item variances of judge ratings. Figure 2 illustrates that $r_{\text{wg}(j)}^{*}$ has the favorable property of linearity, meaning that it will not be affected by increasing scale items. Lindell et al. (1999) suggested that interpretation may be aided by keeping the range of admissible values to those of James et al.'s (1984) *r*_wg_ and *r*_wg(*j*)_ (i.e., 0–1.0). Lindell et al. (1999) pointed out that this could be done by setting the expected random variance, σ_e_^2^, to the maximum possible disagreement, known as maximum dissensus. Maximum dissensus (σ_mv_^2^) is:

(6) $$\sigma_{\text{mv}}^{2}=0.5({{\text{X}}^{2}}_{\text{U}}{+\text{X}}^{2}{}_{\text{L}})-{\left[0.5({\text{X}}_{\text{U}}{+\text{X}}_{\text{L}})\right]}^{2}$$

where X_*U*_ and X_*L*_ are the upper and lower discrete Likert categories, respectively (e.g., “5” and “1” on a five-point scale; Lindell, 2001). Maximum dissensus occurs when all judges are distributed evenly at the scale endpoints, and it can be used in the denominator of the $r_{\text{wg}}^{*}$ or $r_{\text{wg}\left(\text{j}\right)}^{*}$ equations. For example, for multi-item scales:

(7) $$r_{\text{wg}(j)}^{*}=1-(\bar{{S_{\text{x}}}^{2}}/{\sigma_{\text{mv}}}^{2})$$

It is instructive to point out that on a five-point scale, σ_eu_^2^ is 2 and σ_mv_^2^ is 4. Thus, the use of maximum dissensus essentially rescales James et al.'s (1984) *r*_wg_ such that all values of *S*_x_^2^ will result in $r_{\text{wg}}^{*}$ values within the range of 0 and 1.0. This index avoids the problem of non-linearity and corresponding inflation potential of *r*_wg(*j*)_ and addresses the problem of inadmissible values.

### Interpretation

Figure 2 contains functions for $r_{\text{wg}}^{*}$ and $r_{\text{wg}\left(\text{j}\right)}^{*}$ with σ_eu_^2^ and $\sigma_{\text{mv}}^{2}$. Values for $r_{\text{wg}}^{*}$ and $r_{\text{wg}\left(\text{j}\right)}^{*}$will range from −1.0 to 1.0 if the denominator is σ_eu_^2^, wherein a value of 0 is uniform disagreement (i.e., *S*_x_^2^ = σ_eu_^2^) and a value of −1.0 is maximum dissensus (i.e., *S*_x_^2^ = σ_mv_^2^). Note the advantage of $r_{\text{wg}}^{*}$ and $r_{\text{wg}\left(\text{j}\right)}^{*}$ in that information is preserved by assigning a meaningful interpretation to negative values. Values for $r_{\text{wg}}^{*}$ and $r_{\text{wg}\left(\text{j}\right)}^{*}$ will range from 0 to 1.0 when the denominator is σ_mv_^2^, wherein a value of 0.5 is uniform disagreement (i.e., *S*_x_^2^ = σ_eu_^2^), and a value of 0 is maximum dissensus (i.e., $S_{\text{x}}^{2}$ = σ_mv_^2^). Taken together, $r_{\text{wg}}^{*}$ and $r_{\text{wg}(j)}^{*}$ potentially address three drawbacks of James et al.'s (1984) statistics. First, negative values are interpretable by incorporating the concept of maximum dissensus. Second, by using $S_{\text{x}}^{2}$ in the numerator, the multi-item agreement index is not extensively affected by the addition of scale items, which is a major interpretational difficulty of $r_{\text{wg}\left(\text{j}\right)}^{*}$. Third, $r_{\text{wg}}^{*}$ and $r_{\text{wg}(j)}^{*}$ have the further advantage of avoiding inadmissible values that exceed +1.0.

## Further advances on $r_{\text{wg}}^{*}$: disattenuated multi-item $r_{\text{wg}}^{*}$: (*r'*_WG(_*_*j*_*_)_)

### General logic

One of the difficulties with Lindell et al.'s (1999) observed $r_{\text{wg}}^{*}$ statistics, described above, is the use of $\sigma_{\text{mv}}^{2}$ when comparisons between James et al.'s (1984) *r*_wg_ are of interest (Lindell, 2001). The problem lies in the differences in ranges; James et al.'s (1984) *r*_wg_ statistics have admissible values within 0 and +1.0, whereas for $r_{\text{wg}}^{*}$ statistics that use $\sigma_{\text{mv}}^{2}$ the admissible range is from 1.0 to +1.0. Two steps could be taken to remedy this problem. First, as mentioned above, Lindell et al. (1999) observed that $r_{\text{wg}}^{*}$ statistics could be computed with $\sigma_{\text{eu}}^{2}$. This facilitates comparisons, and also allows the researcher to use a multi-item $r_{\text{wg}}^{*}$ that would have similar behavior compared to single-item *r*_wg_. But, a further problem noted by Lindell (2001) is that $r_{\text{wg}}^{*}$ and $r_{\text{wg}\left(\text{j}\right)}^{*}$ with σ_eu_^2^ will be attenuated in comparison to admissible *r*_wg_ and *r*_wg(*j*)_ values. Thus, a second avenue offered by Lindell (2001) to address the relative attenuation of $r_{\text{wg}\left(\text{j}\right)}^{*}$ using $\sigma_{\text{eu}}^{2}$ is an alternative called *r*′_wg(j)_. *r*′_wg(j)_ uses the variance of raters' scale scores on multi-item scales (referred to as *S*_y_^2^, see Table 1 for derivation details):

(8) $${r^{\prime }}_{\text{wg}(j)}=1-({S_{\text{y}}}^{2}/{\sigma_{\text{eu}}}^{2})$$

### Interpretation

Lindell (2001) demonstrated that *r'*_wg(j)_tends to produce larger values than does $r_{\text{wg}\left(\text{j}\right)}^{*}$ using $\sigma_{eu}^{2}$, thereby addressing the issue of attenuation. Otherwise, *r*′_wg(j)_ has the same general interpretation as does $r_{\text{wg}\left(\text{j}\right)}^{*}$, although it might be expected to share the limitation of being correlated with group mean extremity. A further difficulty might involve the need to extensively explain *r'*_wg(j)_ and its logic as reviewers may not be as familiar with this agreement statistic as they are with more frequently employed agreement indices (see Table 1).

## Pooled agreement for subgroups: *r_wg(p)_*

### General logic

As a possible remedy for the problem of inadmissible *r*_wg_ values that fall below 0 or above 1.0, LeBreton et al. (2005) offered *r*_wg(p)_. The rationale is that inadmissible values suggest bimodal response distributions, and the different clusters comprise subgroups. Therefore, separate IRA could be computed for each subgroup, which could then be pooled. Accordingly, *r*_wg(p)_ computes the sample-size weighted average of raters' variance for the two groups, and this value is used in James et al.'s *r*_wg_ or *r*_wg(*j*)_ (see also Table 1). This will effectively remove the possibility of inadmissible values.

### Interpretation

There are a few noteworthy drawbacks involving the use of *r*_wg__(*p*)_. Calculating the pooled *r*_wg(p)_ requires homogeneity of observed variances (e.g., using Fisher's *F*-test; see Table 1), otherwise pooling the variances to calculate the *r*_wg(p)_ may not be justifiable. Another limitation is that these subgroups may be difficult to identify theoretically or *a priori*; thus, capitalization on chance is possible (LeBreton et al., 2005). This can be contrary to purpose as most researchers are interested in a pre-specified set of judges (e.g., team membership). Finally, given that *r*_wg(p)_ has its basis on *r*_wg_ and *r*_wg(*j*)_, *r*_wg(p)_ would share many of the limitation of James et al.'s (1984) statistics. Notwithstanding these limitations, *r*_wg(p)_ does provide a potentially advantageous extension of *r*_wg_ and *r*_wg(*j*)_ for use when subgroups are suspected.

## Average deviation index

### General logic

One major difficulty inherent in *r*_wg_ is the choice of a suitable null distribution. As reviewed above, there is the choice of the rectangular distribution or the maximum dissensus distribution. Moreover, there are other potential distributions, such as skewed bell-shaped distributions, that may more realistically represent null distributions by taking into account factors such as socially-desirable responding or acquiescence tendencies (James et al., 1984; see also discussions by Schmidt and DeShon, 2003; LeBreton and Senter, 2008). Importantly, the selected distribution affects the magnitude of IRA statistics, their interpretation, and comparisons to other IRA statistics. To circumvent difficulties in choosing a null distribution, Burke et al. (1999) offered the average deviation index. The average deviation is calculated by determining the sum of the differences between each rater and the mean rating divided by the number of raters:

(9) $$AD_{M(j)}=\sum \left(|x_{\text{i}}-\bar{x}|\right)/k$$

where *AD*_*M*__(*j*)_ is the average deviation of judges' ratings on a given item, *x*_*i*_ is a judge's rating on the item, $\bar{x}$ is judges' mean rating on the item, and *k* is the number of judges. When there are multiple items:

(10) $$AD_{M(J)}=\sum AD_{M}{}_{\left(j\right)}/J$$

where *AD*_*M*__(J)_ is the average deviation of judges' ratings from the mean judge rating across items, *AD*_*M*__(*j*)_ is the average deviation on a given item, and *J* is the number of scale items. Note that *AD* can be generalized for use with the median, instead of the mean, in order to minimize the effects of outlier or extreme raters.

### Interpretation

The average deviation approach is advantageous as it provides a direct assessment of IRA without invoking assumptions about the null distribution. Moreover, Burke and Dunlap (2002) made useful inroads for determining cutoffs for supporting aggregation, as they attempt to control for the number of Likert response options by suggesting a cutoff criterion of *A*/6 (where *A* is the number of Likert categories; cutoff criteria are discussed further below). On the downside, like *r*_wg_ statistics, the average deviation will be correlated with the group mean such that means closer to the extremities will be negatively related to average deviation values (see Table 1). In addition, whereas some forms of *r*_wg_ can suffer from inadmissible values, AD has the problem of having no standard range whatsoever. Thus, AD values will be difficult to compare across scales with a different numbers of categories.

## Brown and hauenstein's “alternative” estimate of IRA: *a_wg(1)_*

### General logic

Brown and Hauenstein (2005) developed the *a*_wg(1)_ to overcome the limitation of other agreement indices that are correlated with the extremeness of mean ratings. The closer the mean rating is to the scale endpoint (i.e., the extremity of the group mean), the lower the variance in those ratings, and the greater the agreement. This confounds all of the above IRA statistics with the group mean and consequently renders them incomparable across groups with different means. Accordingly, Brown and Hauenstein presented *a*_wg(1)_, which uses, as a null distribution, the maximum possible variance (i.e., maximum dissensus) given a group's mean:

(11) $${S_{\text{mpv}/\text{m}}}^{2}={\left[(\text{H}+\text{L})M-(M^{2})-{\text{H}}^{*}\text{L}\right]}^{*}[k/(k-1)]$$

where *S*_mpv/m_^2^ is the maximum possible variance given *k* raters, *M* is the observed mean rating, and H and L are the maximum and minimum discrete scale values, respectively. Once the maximum possible variance is known, the single-item *a*_wg_ is:

(12) $$a_{\text{wg}}=1-[(2^{*}{S_{x}}^{2})/{S_{\text{mpv}/\text{m}}}^{2}]$$

Note that multiplying *S*_*x*_^2^ by 2 is arbitrary, and is done to give it the same empirical range as James et al.'s (1984) *r*_wg_. For multi-item scales, the single-item *a*_wg_s are averaged:

(13) $$a_{\text{wg}(\text{j})}=\sum a_{\text{wg}(1)}/J$$

### Interpretation

Figure 2 contains *a*_wg_ values for means of 3, 2.5, and 3.5, on a five-point scale. Values of −1.0 indicate maximum dissensus (i.e., judge's ratings are on the scale endpoints as much as possible so as to maximize observed variance, *S*_x_^2^), 0 indicate the observed variance is 50% of the maximum variance (i.e., uniform disagreement), and +1.0 indicate perfect agreement, given the group mean. Note that this is the same interpretation as of the single-item *r*_wg_, except *a*_wg_ is adjusted for the group mean. Moreover, single and multi-item *a*_wg_ are linear functions, thereby enhancing ease of interpretation (see Figure 2). Notice that *a*_wg_ for means departing from the midpoint of the scale are slightly lower, thereby taking into account decreases in maximum dissensus as a result of restricted variance. Finally, *a*_wg_ will not be influenced by sample sizes or number of scale anchors, which are notable additional advantages.

One limitation to Brown and Hauenstein's (2005) *a*_wg_ is that *S*_mpv/m_^2^ cannot be applied when the mean is extreme (e.g., 4.9 on a 5-point scale). This is because *S*_mpv/m_^2^ assumes that at least one rater falls on each scale endpoint, although this is impossible given some extreme means. Thus, there are boundaries in means, outside of which appropriate maximum variance estimates should not be applied (Brown and Hauenstein):

(14) $$\text{Minimum mean with interpretable }a_{\text{wg}}=[L(k-1)+\text{H}]/k$$

(15) $$\text{Maximum mean with interpretable }a_{\text{wg}}=[H(k-1)+\text{L}]/k$$

where L and H are the lowest and highest scale values, and *k* is the number of judges. This is however, a relatively modest limitation because mean ratings falling beyond these boundaries are likely to indicate strong agreement, as values close to the endpoints will only occur when agreement is high. Nevertheless, *a*_wg_ scores exceeding interpretational boundaries cannot be compared at face value to other groups' *a*_wg_s. An additional limitation of *a*_wg_ is that, unlike most other IRA statistics, *a*_wg_ is based on more than a single parameter (e.g., the observed variance, $S_{x}^{2}$). It also includes the mean. As both *S*_x_^2^ and $\bar{x}$ are affected by sampling error, sampling error may have a greater influence on *a*_wg_ than on some other IRA statistics (Brown and Hauenstein). Limitations aside, *a*_wg_ is advantageous because it controls for the mean rating using a mean-adjusted maximum dissensus null distribution and it has a linear function.

Unlike other agreement statistics, *a*_wg_ matches the variances (*S*_x_^2^, *S*_mpv/m_^2^) on whether they employ the unbiased (denominator is *n* − 1) or population-based (denominator is *n*) variance equations. The *r*_wg_ family mixes unbiased and population-based variances (i.e., *S*_x_^2^, σ_eu_^2^, respectively), thereby potentially leading to inflation of *S*_*x*_^2^ as sample sizes decreases (see Brown and Hauenstein, 2005). This results in larger values for the *r*_wg_ family as sample size increases, and, therefore, IRA agreement will almost always be high in large samples (Kozlowski and Hattrup, 1992). Conversely, *a*_wg_ matches the variances by employing sample-based equations for both of *S*_x_^2^ and *S*_mpv/m_^2^, making *a*_wg_ independent of sample size. If population-level data is obtained, controls for sample size can be employed by substituting *k* for *k*−1 in both of *S*_x_^2^ and *S*_mpv/m_^2^ (Brown and Hauenstein; see Table 1).

## Standard deviation

### General logic

The square root of the variance term used throughout the current article, *S*_x_^2^, is the standard deviation, *S*_wg_. As *S*_wg_ is the square root of the average squared deviations from the mean, Schmidt and Hunter (1989) advocated for *S*_wg_ as a straightforward index of IRA around which confidence intervals can be computed. Using the standard deviation addresses problems associated with choosing a null distribution and of non-linearity. The average *S*_wg_ across items can be used in the case of multi-item scales.

### Interpretation

Advantages of using *S*_wg_ as an index of IRA is that it is a common measure of variation, and its interpretation is not complicated by the use of multi-item scales or non-linear functions [see *r*_wg(*j*)_]. However, the *S*_wg_ has not always enjoyed widespread application. It cannot be explicitly compared to random response distributions, and this could be of interest. It also tends to increase with the size of the scale response options, meaning that comparisons across scales are not feasible. Finally, it will also tend to decrease with increases in sample size; thus, it will not be sample-size independent.

## Coefficient of variation

A problem with most IRA statistics reviewed is that they are scale dependent, making comparisons across scales with widely discrepant numbers of Likert response options problematic. With greater numbers of response options, the variance will tend to increase. Thus, the amount of variance (e.g., *S*_x_^2^) could partly depend on scaling, thereby presenting a possible source of contamination for many IRA statistics. One way to address this difficulty is to control for the group mean, because means will typically be larger with greater numbers of response options. One statistic that attempts to address this issue is the coefficient of variation (*CV*_wg_). The *CV*_wg_ indexes IRA by transforming the standard deviation into a variance estimate that is less scale dependent, using the following:

(16) $$CV_{\text{wg}}={\left\{[\sum {\left(x_{\text{i}}-\bar{x}\right)}^{2}]/(n-1)\right\}}^{1/2}/\bar{x}$$

## Multi-item *CV*_wg_ could be computed by averaging *CV*_wg_ over the *J* items.

### Interpretation

**Figure 3:**
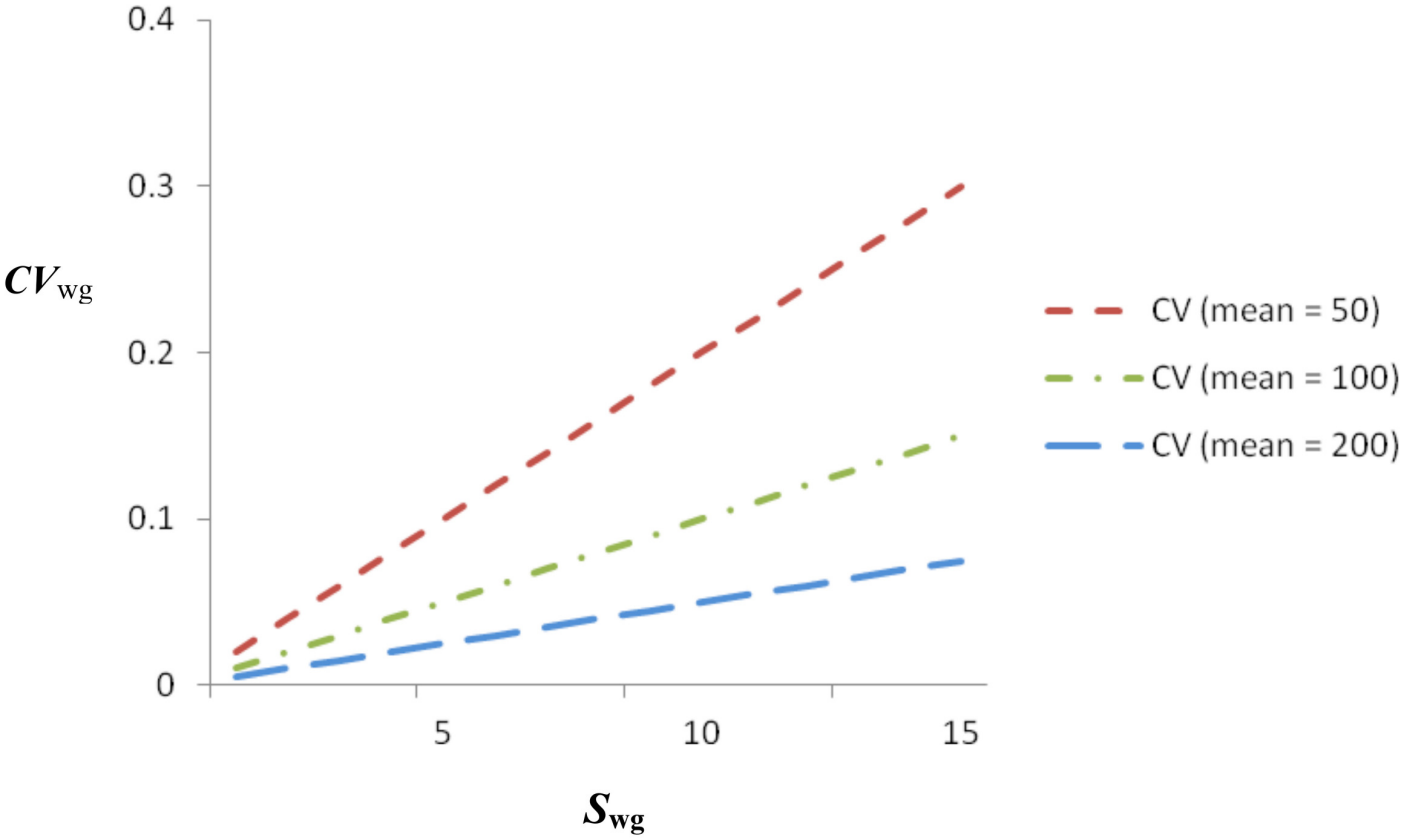
*****CV***_**wg**_ from 0 to 15 within-group standard deviations (***S***_**wg**_) and means of 50, 100, and 200**.

By dividing the standard deviation (the numerator) by the group mean, *CV*_wg_ aims to provide an index of IRA that is not severely influenced by choice of scale, thereby facilitating comparisons of IRA across different scales. For example, the *CV*_wg_ for a sample with a standard deviation of 6 and a mean of 100 would be identical to the *CV*_wg_ for a sample with a standard deviation of 12 and a mean of 200. Figure 3 contains *CV*_wg_ for means equal to 50, 100, and 200 with standard deviations ranging from 0 to 15. An inspection of Figure 3 indicates that the *CV*_wg_ increases faster with increases in standard deviations for low means than for high means, thereby taking into account the difference in variation that may be related to scaling. Thus, the *CV*_wg_ could be helpful in comparing IRA across scales with different numbers of response options. On the other hand, it is only helpful for relative (to the mean) comparisons, and not absolute comparisons (Allison, 1978; Klein et al., 2001). This can be clarified by observing that the addition of a constant to a set of scores will affect the mean and not the standard deviation, making it difficult to offer meaningful interpretations of absolute *CV*_wg_s (but ratio scaling helps; Bedeian and Mossholder, 2000). Another issue is that negative *CV*_wg_ will occur in the presence of a negative mean, but a negative *CV*_wg_ is not theoretically interpretable. Thus, a further requirement is non-negative scaling (Roberson et al., 2007).

## Standards for agreement

What constitutes strong agreement within raters? This is an important question as researchers wishing to employ IRA statistics to support and justify decisions. For example, aggregation of individuals' responses to the group (mean) level may assume a certain level of consensus (Chan, 1998). Or, consensus thresholds may be used in the critical incident technique in job analysis, where performance levels of the employees involved in the incident should be agreed upon by experts (Flanagan, 1954). Identifying a unified set of standards for agreement, however, has proven elusive. Two general approaches to identifying standards for agreement have been suggested: statistical and practical. These are considered briefly below.

### Practical standards

Historically, the emphasis on IRA has been on practical standards or “rules of thumb.” For example, the *r*_wg_ family of statistics has relied on the 0.70 rule of thumb. It is important to acknowledge that the decision to choose 0.70 as the cutoff was based on what amounted to no more than a phone call (see personal communication, February 4th, 1987, in (George, 1990), p. 110; for a discussion, see LeBreton et al., 2003; Lance et al., 2006), and James et al. (1984) likely never intended for this cutoff to be so strongly, and perhaps blindly, adopted. Nevertheless, a perusal of Figures 1, 2 clearly shows why common, rule of thumb standards for any of these statistics are difficult to support. A value of 0.70 has a different meaning for most statistics in the *r*_wg_ family. Further, even within-statistic, different situations may render that statistic incomparable. For example, agreement of 0.70 for an *r*_wg_ based on 10 items vs. an *r*_wg(*j*)_ based on two items is a different agreement benchmark because of the non-linearity. Identification of the null distribution is another influencing factor, as Figure 2 clearly shows that use of $\sigma_{\text{mv}}^{2}$ vs. $\sigma_{\text{eu}}^{2}$ changes the interpretation of any absolute *r*_wg_ value (e.g., 0.70), not to mention other potential null distributions (see LeBreton and Senter, 2008).

As noted by Harvey and Hollander (2004), justification for a cutoff of 0.70 is based on an assumption that agreement is similar to reliability, and reliabilities exceeding 0.70 are preferred. However, reliability is about consistency of test scores, not absolute agreement of test scores. Test scores can be perfectly reliable (consistent) but very distinct in absolute quantities. Reliability can, and should be, approached using Generalizability Theory. G-theory involves the systematic investigation of all sources of consistency and error (Cronbach et al., 1972). For example, O'Neill et al. (2015) identified raters as the largest source of variance in performance ratings, rather than rates or dimensions. Thus, drawing on the 0.70 cutoff from reliability theory is not tenable as this rule of thumb underscores the complexity of reliability. An additional assumption is that a single value (e.g., 0.70) would be meaningfully compared across situations and possibly statistics in the *r*_wg_ family. These assumptions seem untenable, and adopting any standard rule of thumb for agreement involving the entire *r*_wg_ family would appear to be misguided (see Harvey and Hollander, 2004). For many of the same reasons (i.e., incomparability across different situations), there is no clear avenue for setting practical cutoff criteria for *S*_wg_ and *CV*_wg_.

LeBreton and Senter (2008) proposed that standards for interpreting IRA could follow the general logic advanced by Nunnally (1978; see also Nunnally and Bernstein, 1994). Specifically, cutoff criteria should be more stringent when decisions will be highly impactful on the individuals involved (e.g., performance appraisal for administrative decision making). Where applicable, LeBreton and Senter (2008) added that cutoff criteria should consider the nature of the theory underlying aggregation for multilevel research, and the quality of the measure (e.g., newly-established measures may be expected to show lower IRA than do well-established measures). For application to the *r*_wg_ family, the following standards were recommended: 0–0.30 (lack of agreement), 0.31–0.50 (weak agreement), 0.51–0.70 (moderate agreement), 0.71–0.90 (strong agreement), and 0.91–1.0 (very strong agreement). Whereas, these standards will have different implications and meaning for different types of *r*_wg_ and null distributions (consider Figures 1, 2), LeBreton and Senter (2008) proposed the standards for all forms of *r*_wg_. Thus, there is a strong “disincentive” to report versions of *r*_wg_ that will result in the appearance of lower IRA (e.g., using a normal distribution for the null; LeBreton and Senter, p. 836). Nevertheless, they challenged researchers to select the most appropriate *r*_wg_ by using theory (especially in the identification of a suitable null distribution), with the hope that professional judgment will prevail. Future research will be telling with regard to whether or not researchers adopt LeBreton and Senter's (2008) recommended practices.

Turning to other IRA statistics, Burke and Dunlap (2002) suggested that practical significance standards for *AD* could apply the decision rule *A*/6 (where *A* is the number of Likert categories). Thus, for a five point Likert scale 5/6 = 0.83, and *AD* values exceeding 0.83 would be seen as not exhibiting strong agreement. But this decision rule makes two assumptions in its derivation (see Burke and Dunlap for details): (a) the basis is in classical test theory and that interrater reliability should exceed 0.70; and (b) the appropriate null distribution is the rectangular distribution. If these assumptions can be accepted, then the AD has a sound approach for determining cutoffs for practical significance. But if that “null distribution fails to model disagreement properly, then the interpretability of the resultant agreement coefficient is suspect” (Brown and Hauenstein, 2005, p. 166). Elsewhere, Burke et al. (1999) proposed different criteria. They suggested that *AD* should not exceed 1.0 for five- and seven-point scales, and *AD* should not exceed 2.0 for 11-point scales. Finally, it should be noted that Brown and Hauenstein (2005) proposed rules of thumb for *a*_wg_. Specifically, 0–0.59 was considered unacceptable, 0.60–0.69 was weak, and 0.70–0.79 was moderate, and above 0.80 was strong agreement.

### Statistical standards

Identifying standards for IRA using statistical significance testing involves conducting Monte Carlo simulations or random group resampling. For Monte Carlo simulations, the input is the correlation matrix of scale items, the null distribution, and the significance level (see Cohen et al., 2001, 2009; Burke and Dunlap, 2002; Dunlap et al., 2003). Tabled significance values were provided by several researchers (e.g., Dunlap et al., 2003; Cohen et al., 2009). The program *R* contains commands for running Monte Carlo simulations involving *r*_wg_ and *AD* (see Bliese, 2009). The objective is to create a sampling distribution for the IRA statistic with an expected mean and standard deviation, which can be used to generate confidence intervals and significance tests. Random group resampling involves constructing a sampling distribution by repeatedly sampling and forming random groups from observations in the observed data set, and comparing the significance of the mean difference in within-group variances of the observed distribution and the randomly generated distribution using a *Z*-test (Bliese et al., 2000; Bliese and Halverson, 2002; Ludtke and Robitzsch, 2009), for which commands are available in *R*. Thus, significance testing of the *S*_wg_ is possible through the random-group resampling approach. Similar logic could be applied to test the significance of $r_{\text{wg}}^{*}$, a_wg_, and *CV*_wg_, although existing scripts for running these tests may be more difficult to find.

Statistical significance testing of IRA statistics has its advantages. Cutoff criteria are relatively objective, thereby potentially reducing misuse by relying on inappropriate or arbitrary rules of thumb (see below). But, statistical significance does not appear to have been widely implemented. One reason might be because of the novelty of the methods for doing so, and the need to understand and implement commands in *R*, for example. Another reason might be because statistical agreement might be difficult to reach in many commonly-encountered practical situations. Specifically, many applications will involve three to five raters, yet *r*_wg(*j*)_ needs to be in the range of at least 0.75 and AD would have to fall below 0.40 (Burke and Dunlap, 2002; Cohen et al., 2009) in order to reject the null hypothesis of no agreement. Indeed, Cohen et al. reported that groups with low sample sizes rarely reached levels of statistical significance that would allow the hypothesis of no statistical agreement to be rejected. If statistical significance testing is treated as a hurdle against which agreement must be passed in order for further consideration of the implicated variables, there is potential to interfere with advancement of research involving low (but typical) sample sizes. This may not always be the most desirable application of IRA, and, not surprisingly, practical standards have tended to be most common.

### Current best practice in judging agreement levels

It is important to acknowledge the two divergent purposes of practical and statistical approaches to judging agreement. Practical cutoffs provide decision rules about whether or not agreement seems to have exceeded a minimum threshold in order to justify a decision. Examples of such decisions include aggregation of lower-level data to higher-level units, retention of critical incidents in job analyses, and for assessing whether frame-of-reference training has successfully “calibrated” raters. The use of practical cutoff criteria in these decisions implies that a certain level of agreement is needed in order to make some practical decision in light of the agreement qualities of the data (Burke and Dunlap, 2002).

Statistical standards are not focused on the absolute level of agreement so much as they are concerned with drawing inferences about a population given a sample. Statistical agreement tests the likelihood that the observed agreement in the sample is greater than what would be expected by chance at a certain probability value (e.g., *p* < 0.05). It involves making inferences about whether the sample was most likely drawn from a population with chance levels of agreement vs. systematic agreement. For example, a set of judges could be asked to rate the job relevance of a personality variable in personality oriented job analysis (Goffin et al., 2011). If agreement is not significant for a particular variable, it would suggest that there is no systematic agreement in the population of judges (Cohen et al., 2009). Notice that this differs from practical significance, which would posit a cutoff, above which agreement levels would be considered adequate for supporting the use of the mean rating as an assessment of the job relevance of the trait (e.g., O'Neill et al., 2011).

Statistical agreement raises issues of power and sample size. Specifically, in small samples statistical agreement will be more difficult to reach than in large samples. Accordingly, outcomes of whether agreement is strong or not may depend on whether one focuses on statistical or practical decision standards, and in large samples, statistical agreement alone should not sufficiently justify aggregation (Cohen et al., 2009). The key point, however, is that statistical significance testing is for determining whether the agreement level for a particular set of judges exceeds chance levels. Practical agreement is about absolute levels of agreement in a sample, which could be seen as strong even for non-significant agreement when sample sizes are low.

In light of the above discussion, it is clear that more research is needed in order to identify defensible and practical approaches for judging IRA levels. Best practice recommendations for the interim would involve reporting several IRA statistics, ideally from different families, in order to provide a balanced perspective on IRA. Practical significance levels could be advanced *a priori* using suggestions described above (e.g., Burke and Dunlap, 2002; Brown and Hauenstein, 2005; LeBreton and Senter, 2008) in order to identify cutoffs for making decisions. Statistical significance would be employed only when inferences about the population are important and when a power analysis suggests sufficient power to detect agreement, although practical standards should also be considered especially when power is very high. Thus, a researcher or practitioner might place little emphasis on statistical significance when he or she is not concerned about generalizing to the population, and when there are very few judges the researcher might be advised to consider a less stringent significance level (e.g., α = 0.10). Importantly, when evaluating agreement in a set of judges, the focus is typically not on whether the sample was drawn from a population with chance or systematic agreement, but whether there is a certain practically meaningful level of agreement. Thus, in many cases practical significance might be most critical.

Interpretations of practical agreement should probably not be threshold-based, all-or-none decision rules applied to a single statistic [e.g., satisfactory vs. unsatisfactory *r*_wg(*j*)_]. This is how statistics can be misused to support a decision (see Biemann et al., 2012). Rather, reporting the values from several IRA statistics along with proposed practical standards of agreement reviewed here will provide some evidence of the quality of the ratings, which can be considered in the context of other important indices that also reflect data quality (e.g., reliability, validity). An overall judgment can then be advanced and the reader (including the reviewer) will also have the necessary information upon which to form his or her own judgment. This procedure fits well within the spirit of the unitary perspective on validity (Messick, 1991; Guion, 1998), which suggests that validity involves an expert judgment on the basis of all the available reliability and validity evidence regarding a construct. It would seem that IRA levels should be considered in the development of this judgment, but it may not be productive to always require an arbitrary level of agreement to support or disconfirm the validity of a measure in a single study. In any case, consequential validity (Messick, 1998, 2000) should be kept in mind, and more research examining the consequences, implications, and meaning of various standards for IRA is needed.

## Conclusion

IRA statistics are critical to justification of aggregation in multilevel research, but they are also frequently applied in job analysis, performance appraisal, assessment centers, employment interviews, and so forth. Importantly, IRA offers a unique perspective from reliability because reliability deals with consistency of ratings and agreement deals with the similarity of absolute levels of ratings. IRA has the added advantage of providing one estimate per set of raters—not one estimate for the sample as is the case with reliability. This feature of IRA can be helpful for diagnostic purposes, such as identifying particular groups with high or low IRA.

Despite the prevalence of IRA, there is the problem that articles considering IRA statistics tend to be heavy on the technicals (e.g., Lindell and Brandt, 1999; Cohen et al., 2001), and this might be a reason why *r*_wg_, with its widely known limitations (e.g., see Brown and Hauenstein, 2005), appears to persevere as the leading statistical choice for IRA. Indeed, a recent review suggested that a lack of a sound understanding of IRA statistics may have led to some misuses (see Biemann et al., 2012). Thus, despite the many alternatives offered (e.g., $r_{\text{wg}}^{*}$, *AD, a*_wg_, *CV*_wg_), they may not receive full consideration because accessible, tractable, and non-technical resources describing each within a framework that allows for simple contrasting is not available. LeBreton and Senter (2008) provided solid coverage, but it was mainly with respect to multilevel aggregation issues and not directly applicable to other purposes (e.g., job analysis).

The current article aims to fill a gap in earlier research by offering an introductory source, intended to be useful for scholars with a wide range of backgrounds, in order to facilitate application and interpretation of IRA statistics. Through a comparative analysis regarding eight IRA statistics, it appears that these statistics are not interchangeable and that they are differentially affected by various contextual details (e.g., number of Likert response options, number of judges, number of scale items). The goal of the article is to facilitate critical and appropriate applications of IRA in the future, offer a foundation for tackling the more technical sources currently available, and make suggestions regarding best practices in light of the insights gleaned through the review. It is proposed that researchers interpret IRA levels with respect to the situation and best-practice recommendations for practical and statistical standards in the literature, as reviewed here. Because of the unique limitations of each statistic, it is probably safe to conclude that more than one statistic should always be reported. In submissions where this has been ignored, reviewers should request the author to report additional agreement statistics, ideally from other IRA families. Consistent with the unitary perspective on validity, it is suggested that judgments regarding the adequacy of the ratings rely on evidence of IRA in conjunction with additional statistics that shed light on the quality of the data (e.g., reliability coefficients, criterion validity coefficients). Regarding agreement standards, it would seem advisable to evaluate a given IRA statistic using appropriate *a priori* practical cutoffs and statistical criteria, depending on the purpose of assessing agreement levels. What we need to avoid is misuses of agreement statistics and adoption of inappropriate or misleading decision rules. This critical review aims to provide tools to help researchers and practitioners avoid these problems.

## Author contributions

The author confirms being the sole contributor of this work and approved it for publication.

### Conflict of interest statement

The author declares that the research was conducted in the absence of any commercial or financial relationships that could be construed as a potential conflict of interest.
